# Cerebral Blood Flow Pattern Changes in Unilateral Sudden Sensorineural Hearing Loss

**DOI:** 10.3389/fnins.2022.856710

**Published:** 2022-03-09

**Authors:** Yue Chen, Haimei Li, Bing Liu, Wenwen Gao, Aocai Yang, Kuan Lv, Hui Xia, Wenwei Zhang, Hongwei Yu, Jian Liu, Xiuxiu Liu, Yige Wang, Honglei Han, Guolin Ma

**Affiliations:** ^1^Department of Radiology, China-Japan Friendship Hospital, Beijing, China; ^2^Department of Radiology, Fuxing Hospital, Capital Medical University, Beijing, China; ^3^Institute of Electrical Engineering, Chinese Academy of Sciences, Beijing, China; ^4^Department of Ultrasound Diagnosis, China-Japan Friendship Hospital, Beijing, China; ^5^Department of Otolaryngology, China-Japan Friendship Hospital, Beijing, China

**Keywords:** sudden sensorineural hearing loss, cerebral blood flow, arterial spin labeling, brainnetome atlas, magnetic resonance imaging

## Abstract

**Objective:**

This study analyzed the differences in the cerebral blood flow (CBF) between unilateral Sudden Sensorineural Hearing Loss (SSNHL) patients and healthy controls (HCs). We also investigated CBF differences in auditory-related areas in patients with left- and right-sided SSNHL (lSSNHL and rSSNHL) and HCs. We further explore the correlation between unilateral SSNHL characteristics and changes in the CBF.

**Methods:**

36 patients with unilateral SSNHL (15 males and 21 females, 40.39 ± 13.42 years) and 36 HCs (15 males and 21 females, 40.39 ± 14.11 years) were recruited. CBF images were collected and analyzed using arterial spin labeling (ASL). CereFlow software was used for the post-processing of the ASL data to obtain the CBF value of 246 subregions within brainnetome atlas (BNA). The Two-sample *t*-test was used to compare CBF differences between SSNHL patients and HCs. One-way ANOVA or Kruskal-Wallis test was used to compare the CBF difference of auditory-related areas among the three groups (lSSNHL, rSSNHL, and HCs). Then, the correlation between CBF changes and specific clinical characteristics were calculated.

**Results:**

The SSNHL patients exhibited decreased CBF in the bilateral middle frontal gyrus (MFG, MFG_7_1 and MFG_7_3), the contralateral precentral gyrus (PrG, PrG_6_3) and the bilateral superior parietal lobule (SPL, bilateral SPL_5_1, SPL_5_2, and ipsilateral SPL_5_4), *p* < 0.0002. Compared with HCs, unilateral SSNHL patients exhibited increased rCBF in the bilateral orbital gyrus (OrG, OrG_6_5), the bilateral inferior temporal gyrus (ITG, contralateral ITG_7_1 and bilateral ITG_7_7), *p* < 0.0002. lSSNHL showed abnormal CBF in left BA21 caudal (*p* = 0.02) and left BA37 dorsolateral (*p* = 0.047). We found that the CBF in ipsilateral MFG_7_1 of SSNHL patients was positively correlated with tinnitus Visual Analog Scale (VAS) score (*r* = 0.485, *p* = 0.008).

**Conclusion:**

Our preliminary study explored CBF pattern changes in unilateral SSNHL patients in auditory-related areas and non-auditory areas, suggesting that there may exist reduced attention and some sensory compensation in patients with SSNHL. These findings could advance our understanding of the potential pathophysiology of unilateral SSNHL.

## Introduction

Sudden Sensorineural Hearing Loss (SSNHL) is defined as a hearing loss of over 30 dB in three sequential frequencies in the pure-tone audiogram within 72 h, and is considered as an otologic emergency (Stachler et al., 2012). SSNHL is often accompanied by tinnitus and vertigo, which affects quality of life of patients, leading to anxiety and depression (Fusconi et al., 2012). Many causes of SSNHL have been proposed, including viral infection, neoplasms, trauma, ototoxicity, autoimmune diseases, and developmental anomalies, but the mechanisms of SSNHL have not yet been clarified. It is difficult to identify the exact cause of SSNHL by routine clinical examinations (Jung da et al., 2016). At present, steroid therapy is the main treatment method for SSNHL. But some SSNHL patients do not respond to conventional treatment, which leads to a low long-term quality of life (Liu et al., 2020). Previous studies have reported that hearing loss induced brain structural changes, brain functional changes and reorganization of functional cerebral networks. From the brain structural perspective, brain morphological changes could be induced by unilateral hearing loss was found, including decreased gray matter volume in temporal/ precuneus/ cingulate and parahippocampal gyrus (Yang et al., 2014). From the functional perspective, a functional magnetic resonance imaging (fMRI) study found the changes of default mode networks (DMN) in patients with long-term unilateral SSNHL might be associated with cognitive ability of patients (Zhang et al., 2015). Another resting-state fMRI found that there existed a shift toward small-worldization in unilateral SSNHL patients functional connectome (Xu et al., 2016). A widespread functional reorganization was found in SSNHL, not only in auditory regions (Minosse et al., 2020). Similarly, a diffusion tensor imaging (DTI) study with graph theoretical analysis found that altered nodal centralities in brain regions involved the auditory network and non-auditory network, including visual network, attention network, DMN, sensorimotor network, and subcortical network in SSNHL patients in the acute period (Zou et al., 2021). A study demonstrated cortical perfusion pattern changes in age-related hearing loss (Ponticorvo et al., 2019). All in all, structural and functional changes were found not only in the auditory-related areas, but also in the non-auditory areas in the above studies. Until now, few studies shed light on the cerebral blood flow (CBF) pattern changes in unilateral SSNHL patients. The changes of CBF following SSNHL remain largely unknown.

Arterial spin labeling (ASL) is an MRI technique used to non-invasively measure CBF by magnetically labeling the arterial water (Haller et al., 2016). Regional brain CBF is tightly coupled with regional metabolism and neuronal activity (Li et al., 2020). This study aimed to use three-dimensional pseudo-continuous (3D-pCASL) to detect regional CBF pattern changes in unilateral SSNHL patients by region of interest (ROI) analysis. We analyzed the differences in the CBF between unilateral SSNHL patients and healthy controls (HCs). We also investigated the CBF differences in auditory-related areas in patients with left- and right-sided SSNHL (lSSNHL and rSSNHL) and HCs. We further explore the correlation between unilateral SSNHL characteristics and changes in the CBF. We hope our results will help to characterize unilateral SSNHL pathophysiology.

## Materials and Methods

### Subjects

Thirty-six patients (19 left-sided SSNHL, 17 right-sided SSNHL) were enrolled in this study. Among them, 13 patients were recruited at the outpatient otorhinolaryngology Department of China-Japan Friendship Hospital, and 23 patients were recruited at Fuxing Hospital affiliated to Capital Medical University. The inclusion criteria for patients were: (1) diagnosed as unilateral SSNHL, according to the Sudden Hearing Loss Clinical Practice Guideline of the American Academy of Otolaryngology-Head and Neck Surgery Foundation (Stachler et al., 2012); (2) SSNHL duration time ≤ 3 month; (3) age < 70 years, (4) being right-handedness. The exclusion criteria for patients were: (1) with bilateral SSNHL; (2) have contraindications to MRI examination; (3) suffer from Ménière’s disease, hyperacusis, and history of neurological disorders, such as brain trauma and strokes. Thirty six healthy controls (HC) were recruited from the society. The SSNHL patients and HCs were group-matched in terms of age, sex, and education. This study was approved by the Ethics Committee of China-Japan Friendship hospital and Fuxing Hospital, and informed consent of all the participants was obtained.

### Clinical Data

Demographic information was collected prior to the MRI scan. Hearing thresholds were determined by pure tone audiometry to determine the hearing loss degree. Pure tone average (PTA) value was calculated by averaging the hearing thresholds at 0.5, 1, 2, and 4 kHz. 28 SSNHL patients were accompanied by tinnitus, and we finally obtained the tinnitus questionnaire results from them. The tinnitus loudness was measured by Visual Analogue Scale (VAS) (Mores et al., 2019). The severity of tinnitus was assessed by Tinnitus Handicap Inventory (THI) (Meng et al., 2012). In addition, we evaluated symptoms of depression and anxiety of SSNHL patients according to the Self-Rating Depression Scale (SDS) (Zung, 1972) and Self-Rating Anxiety Scale (SAS) (Zung, 1971). However, as the overall scores of SDS and SAS for SSNHL patients were less than 50, none of them had anxiety or depression.

### Magnetic Resonance Imaging Data Acquisition

13 unilateral SSNHL patients and all 36 HCs were scanned using a 3.0-T MRI scanner (Discovery MR750 scanner, GE Medical Systems, United States) with an 8-channel phased-array head coil in China-Japan Friendship Hospital. 3D-pCASL using the parameters as follows: TR = 4,817 ms, TE = 14.6 ms, slice thickness = 4 mm, slice spacing = 4 mm, flip angle = 111°, post label delay = 1,525 ms, voxel size = 1.875 × 1.875 mm^2^.

The other 23 unilateral SSNHL patients were scanned using a 3.0-T MRI scanner (Discovery MR750w scanner, GE Medical Systems, United States) in Fuxing Hospital. ASL images were obtained with 3D-pCASL using the parameters as follows: TR = 4,640 ms, TE = 10.7 ms, slice thickness = 4 mm, slice spacing = 4 mm, flip angle = 111°, post label delay = 1,525 ms, voxel size = 1.875 × 1.875 mm^2^. All the participants were given ear plugs to reduce scanner noise, and foam paddings were given to restrict head motion.

### Data Processing

**FIGURE 1:**
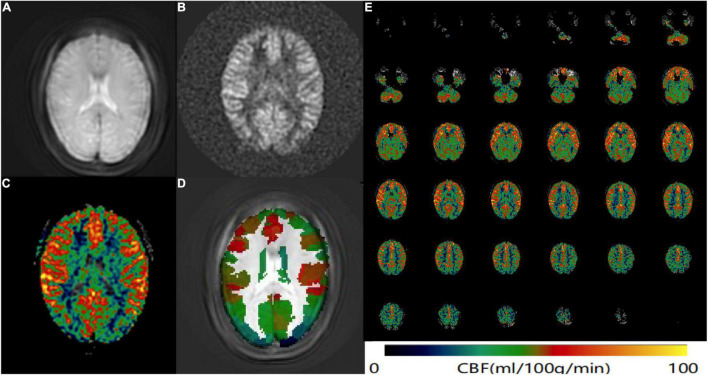
ASL data processing workflow. Convert the **(A)** 3D ASL proton density images and **(B)** perfusion weighted images into the CBF map of cerebral perfusion for each subject. **(C)** The CBF map was normalized to the MNI space and then the BNA was overlaid. **(D)** The average CBF value of each brain region from BNA was obtained. **(E)** An example CBF map of a female SSNHL patient calculated by Cereflow software.

CereFlow software (An-Image Technology Co., China) was used to process ASL data, the steps were as follows: (1) convert the 3D ASL perfusion weighted images and proton density images (generated from GE MR scanner) into the CBF map of cerebral perfusion for each subject by using the simplified one compartment model (St Lawrence and Wang, 2005). (2) normalize the CBF map to the Montreal Neurological Institute (MNI) space with intensity-based image registration where the MNI152 brain template was used as a fixed image, and transform each subject’s image as the motion image to match the fixed image. (3) then the Brainnetome Atlas (BNA) (Fan et al., 2016) was overlaid. (4) the average CBF value of each brain region from BNA was finally obtained (Figure 1).

### Calculation of rCBF

We considered that ASL data acquired from different MR scanners and the individual variance of subjects could affect the study results. The normalized cerebral blood flow value rCBF was calculated for further statistical analysis:


rCBF=CBF÷mCBF

Where CBF is the average CBF value of each brain region in BNA, and mCBF is the average CBF value of the whole brain.

### rCBF in Auditory-Related Areas Analysis

**FIGURE 2:**
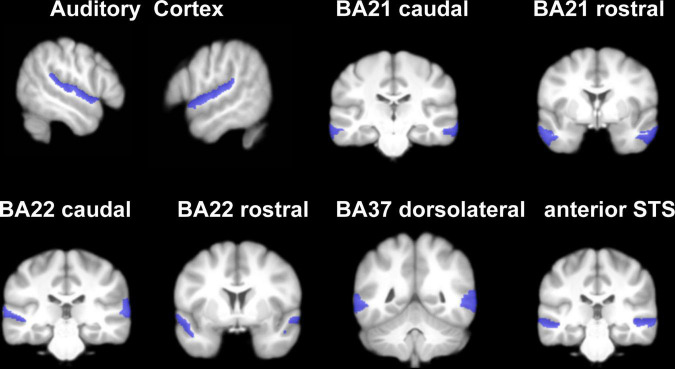
Anatomic representation of the auditory-related areas ROIs within the Brainnetome atlas.

Several studies have confirmed changed neuronal activity of the auditory cortex in SSNHL patients (Minosse et al., 2020; Zou et al., 2021). Considering the lateralization effects of auditory cortex (Tervaniemi and Hugdahl, 2003), we investigated the CBF differences of auditory-related areas among lSSNHL, rSSNHL, and HCs. We conducted an ROI analysis of auditory-related areas for further analysis. Auditory related areas of both hemispheres with in BNA were divided into the following ROIs: auditory cortex (BA41/42; TE1.0 and TE1.2), Wernicke’s area (BA22 caudal and rostral) and associative auditory areas (BA21 caudal and rostral; BA37 dorsalateral; and the anterior part of the Superior Temporal Sulcus) (Martín-Fernández et al., 2021; Figure 2).

### Statistical Analysis

#### Demographic and Clinical Data Analysis

Differences in demographic data between the SSNHL and HC group were analyzed using Two-sample *t*-test and Chi-square test in SPSS 26.0 software (Chicago, IL, United States).

#### Group rCBF Differences: Sudden Sensorineural Hearing Loss vs. Healthy Controls

For more rigorous statistical analysis, the right side of rSSNHL matched controls (17 cases) was defined as the ipsilateral (affected side), and the left side of lSSNHL matched controls (19 cases) was defined as the ipsilateral. Then, the other side was defined as contralateral in this study. rCBF difference between two groups (all the SSNHL patients and HCs) were compared by Two-sample *t*-test, corrected with the Bonferroni method, 246 comparisons (for there were 246 subregions in BNA). *P* < 0.0002 was considered statistically significant.

#### Group rCBF Differences in Auditory-Related Areas: lSSNHL vs. rSSNHL vs. Healthy Controls

One-way ANOVA or Kruskal-Wallis test was used to compare the rCBF difference of each auditory ROI among the three groups (lSSNHL, rSSNHL, and HCs), followed by *post hoc* intergroup comparisons (Bonferroni correction). *P* < 0.05 was considered statistically significant.

#### Correlation Analysis

We used Pearson correlation analysis to calculate the relationships between abnormal rCBF and clinical characteristic data. Partial correlations were calculated after correction for age and gender. *P* < 0.05 was considered statistically significant.

## Results

### Demographics and Clinical Characteristics: Sudden Sensorineural Hearing Loss vs. Healthy Control

**FIGURE 3:**
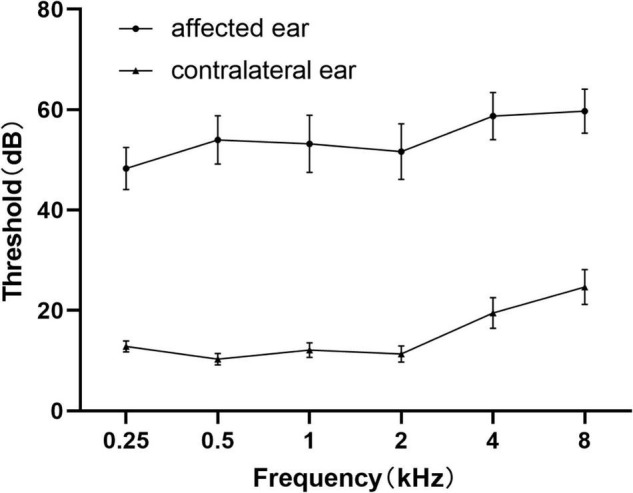
Average pure tone audiograms for patients with SSNHL averaged over both ears at different frequencies. Data are presented as mean ± SEM. SEM, standard error of mean.

Demographics and clinical characteristic data of all the unilateral SSNHL patients and healthy controls were summarized in Table 1 and Figure 3. No significant differences were found in terms of age, gender, education level.

**TABLE 1:** Demographics and clinical characteristics of unilateral SSNHL patients and HC.

	SSNHL (*n* = 36)	HCs (*n* = 36)	*p*-value
Age (years)	40.39 ± 13.42	40.39 ± 14.11	1.000
Gender (M)	15 (41.67%)	15 (41.67%)	1.000
Education level (years)	15.50 ± 3.39	15.42 ± 3.90	0.923
Handedness (R)	36 (100%)	36 (100%)	–
Duration of hearing loss (day)	16.11 ± 24.41	–	–
Tinnitus	28 (77.8%)	–	–
PTA of affected ear (dB)	54.68 ± 28.71	–	–
VAS score	4.86 ± 2.05	–	–
THI score	44.83 ± 25.84	–	–
SAS score	32.40 ± 8.03	–	–
SDS score	36.53 ± 7.24	–	–

*PTA, Pure tone average. VAS, Visual Analog Scale. THI, Tinnitus Handicap Inventory. SAS, Self-Rating Anxiety Scale. SDS, Self-Rating Depression Scale. –, not measured.*

### Group rCBF Differences: Sudden Sensorineural Hearing Loss vs. Healthy Control

**FIGURE 4:**
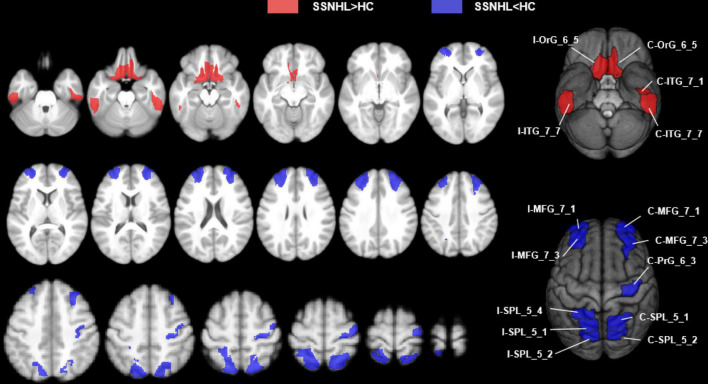
Abnormal CBF subregions in patients with unilateral SSNHL compared with healthy controls. Red, SSNHL (rCBF) > HC (rCBF); Blue, SSNHL (rCBF) < HC (rCBF). I, ipsilateral; C, contralateral; MFG, middle frontal gyrus; PrG, Precentral Gyrus; SPL, superior parietal gyrus; ITG, inferior temporal gyrus; OrG, orbital gyrus.

**FIGURE 5:**
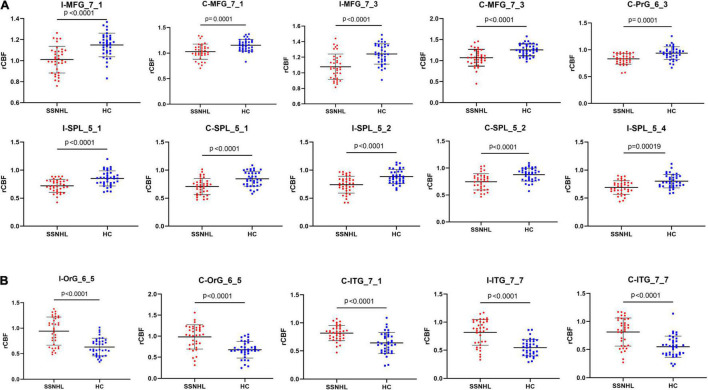
Comparisons of rCBF values between healthy controls and patients with SSNHL. **(A)** Decreased CBF subregions in patients with unilateral SSNHL compared with healthy controls. **(B)** Increased CBF subregions in patients with unilateral SSNHL compared with healthy controls. Data was mean ± SD. I, ipsilateral; C, contralateral; MFG, middle frontal gyrus; PrG, Precentral Gyrus; SPL, superior parietal gyrus; ITG, inferior temporal gyrus; OrG, orbital gyrus; SD, standard deviation.

The SSNHL patients exhibited decreased rCBF in the bilateral middle frontal gyrus (MFG_7_1 and MFG_7_3), the contralateral precentral gyrus (PrG_6_3) and the bilateral Superior Parietal Lobule (bilateral SPL_5_1, SPL_5_2 and ipsilateral SPL_5_4), *p* < 0.0002 (Figures 4, 5A).

Compared with healthy controls, unilateral SSNHL patients exhibited increased rCBF in the bilateral orbital gyrus (OrG_6_5), the bilateral inferior temporal gyrus (contralateral ITG_7_1 and bilateral ITG_7_7), *p* < 0.0002 (Figures 4, 5B).

### Group rCBF Differences: lSSNHL vs. rSSNHL vs. Healthy Control

**FIGURE 6:**
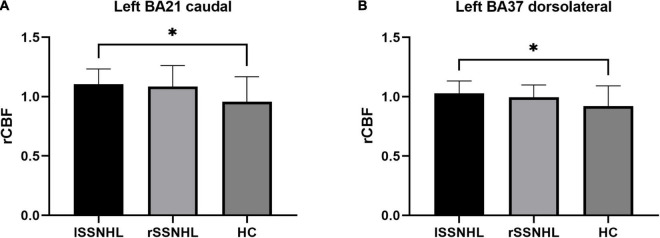
Comparisons of auditory ROIs rCBF values among the three groups (lSSNHL, rSSNHL and HCs). lSSNHL showed increased rCBF in auditory-related areas **(A)** left BA21 caudal (*p* = 0.02) and **(B)** left BA37 dorsolateral (*p* = 0.047) compared to HC. *p < 0.05.

lSSNHL showed abnormal rCBF in left BA21 caudal (*p* = 0.02, One-way ANOVA, Bonferroni correction) and left BA37 dorsolateral (*p* = 0.047, Kruskal-Wallis test, Bonferroni correction) compared to HC. There were no significant differences in the rCBF between the rSSNHL group and HC group or lSSNHL group and rSSNHL group (Figure 6).

### Correlation Analysis

**FIGURE 7:**
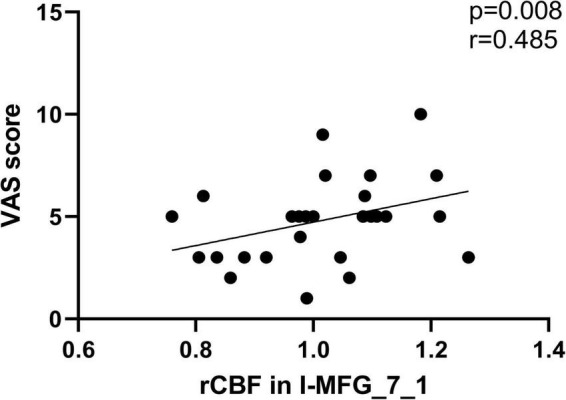
The significant correlations between the CBF changes and the clinical data in unilateral SSNHL patients. CBF in ipsilateral MFG_7_1 of SSNHL patients was positively correlated with VAS score (*r* = 0.485, *p* = 0.008).

We found that the CBF in ipsilateral MFG_7_1 of SSNHL patients was positively correlated with VAS score (*r* = 0.485, *p* = 0.008) (Figure 7).

## Discussion

In this study, the CBF changes in patients with unilateral SSNHL, as well as the relationship between the altered CBF and the clinical characteristics was explored. To the best of our knowledge, this study is the first to investigate the CBF changes in unilateral SSNHL patients using 3D-pCASL. We observed the CBF changes in patients with SSNHL in non-auditory areas, such as visual-related (bilateral ITG) and attention-related areas (bilateral dorsolateral prefrontal cortex, dlPFC) compared with the control. lSSNHL patients showed abnormal CBF in auditory-related areas in this study, whereas rSSNHL patients did not.

Unilateral SSNHL patients exhibited increased CBF in visual-related areas (ITG). The ventral part of temporal cortices, which belongs to the ventral stream of the visual system, are involved in the perception in the recognition of object and human faces features, and scenes (Hickok and Poeppel, 2007). When the central auditory system input is reduced, other sensory inputs would be enhanced correspondingly (Lomber et al., 2010). Previous fMRI studies demonstrated that there may exist transmodal neural change in the visual and sensory system in patients with sensorineural hearing loss (SNHL), which reflects the compensation of brain function (Lomber et al., 2010; Chen et al., 2020). In this study, CBF increase in visual-related areas, suggesting that there may be similar visual compensation in patients with SSNHL. In this study, we also found the increased CBF in bilateral OrG. The OrG belongs to the ventromedial prefrontal cortex, and involves in emotional management and guidance of the concentration of attention (Hiser and Koenigs, 2018; Kuusinen et al., 2018). We speculate that the increased CBF in bilateral OrG might be caused by the emotional reaction of patients with SSNHL.

Unilateral SSNHL patients exhibited decreased CBF in attention-related areas (bilateral dlPFC), primary motor cortex (PrG) and the associative somatosensory cortex (bilateral SPL). dlPFC involves in multi-sensory integration (Fuster, 2000), goal-driven attention (Jones and Graff-Radford, 2021), and as a core node in the executive control network (ECN) (Shen et al., 2020). Attention effects were reduced by the dlPFC lesions (Knight et al., 1981). A multimodal MRI Study indicated that the dlPFC plays an important role in the recruitment of the auditory area into cross-sensory processing in long-term bilateral SNHL patients (Luan et al., 2019). A voxel-based morphometry (VBM) analysis revealed gray matter changes in the SFG and MFG, suggesting a decreased use of ECN in chronic hearing loss patients (Husain et al., 2011). Several studies found the reduction of cortical thickness in SPL of children with SNHL (Shiohama et al., 2019; Qu et al., 2020), which is involved in processing motion stimuli (Qu et al., 2020) and cognitive control (Job et al., 2020). An age-related hearing loss ASL MRI study found the trend of reduced averaged perfusion involved multiple extra-auditory regions in the parietal and prefrontal cortex (Ponticorvo et al., 2019). Unilateral hearing loss causes the reduced attention of SSNHL patients in noisy environment by the deterioration of auditory processing, including speech perception and understanding (Ponticorvo et al., 2019). We speculate that reduced attention of SSNHL patients was related to CBF changes in the parietal and prefrontal lobes. In addition, we also explored the CBF differences of auditory-related cortex among the three groups (lSSNHL, rSSNHL, and HCs). We found that the increased CBF in left BA21 cadual and BA37 dorsolateral in patients with left-sided SSNHL, indicates that there might be a “superactivation” phenomenon in auditory-related areas (Xia et al., 2017). The same phenomenon was not found in patients with right-sided SSNHL, probably due to lateralization and the asymmetry in auditory processing (Tervaniemi and Hugdahl, 2003). The CBF in ipsilateral MFG_7_1 of SSNHL patients was positively correlated with VAS score tinnitus loudness. An fMRI study showed that the abnormal intensity of brain functional connectivity in patients with chronic tinnitus mainly occurred in the non-auditory brain region, especially in the prefrontal cortex (Araneda et al., 2018). Tinnitus may be produced to reduce perceptual uncertainty caused by peripheral auditory deafferentation (Morcom and Friston, 2012), which can explain the reason most SSNHL patients in this study accompanied by tinnitus.

There were several limitations in this study. First and foremost, the ASL data was acquired using different MR scanners, though we calculated rCBF to minimize the effects, the effects can’t be avoided completely. Second, this is a cross-sectional study, and the sample size was small, we first compared the CBF difference between all the SSNHL patients and HCs, then we only investigated CBF differences in auditory-related areas in patients with lSSNHL and rSSNHL. In the future, the CBF changes of all the subregions in patients with lSSNHL and rSSNHL can be studied separately. Third, we cannot completely prevent subjects from hearing some sounds during MRI scans, although we attempted to minimize scanner noise through earplugs in this study.

## Conclusion

Our preliminary study explored CBF pattern changes in unilateral SSNHL patients in auditory-related areas and non-auditory areas, suggesting that there may exist reduced attention and some sensory compensation in patients with SSNHL. CBF changes in ipsilateral MFG_7_1 may influence the tinnitus loudness of SSNHL patients. These findings could advance our understanding of the potential pathophysiology of unilateral SSNHL.

## Data Availability Statement

The original contributions presented in the study are included in the article/supplementary material, further inquiries can be directed to the corresponding author/s.

## Ethics Statement

The studies involving human participants were reviewed and approved by the Ethics Committee of China-Japan Friendship Hospital and Fuxing Hospital. The patients/participants provided their written informed consent to participate in this study.

## Author Contributions

YC and HL acquired and analyzed the ASL data, and drafted the manuscript. BL, WG, HX, WZ, and JL searched and managed the literature. AY, KL, XL, YW, and HY did the MRI scanning. GM and HH designed the study and revised the manuscript. All authors contributed to the article and approved the submitted version.

## Conflict of Interest

The authors declare that the research was conducted in the absence of any commercial or financial relationships that could be construed as a potential conflict of interest.

## Publisher’s Note

All claims expressed in this article are solely those of the authors and do not necessarily represent those of their affiliated organizations, or those of the publisher, the editors and the reviewers. Any product that may be evaluated in this article, or claim that may be made by its manufacturer, is not guaranteed or endorsed by the publisher.

## References

[B1] Araneda R. Renier L. Dricot L. Decat M. Ebner-Karestinos D. Deggouj N. (2018). A key role of the prefrontal cortex in the maintenance of chronic tinnitus: An fMRI study using a Stroop task. *Neuroimage Clin.* 17 325–334. 10.1016/j.nicl.2017.10.029 29159044 PMC5675730

[B2] Chen J. Hu B. Qin P. Gao W. Liu C. Zi D. (2020). Altered Brain Activity and Functional Connectivity in Unilateral Sudden Sensorineural Hearing Loss. *Neural Plast.* 2020:9460364. 10.1155/2020/9460364 33029130 PMC7527900

[B3] Fan L. Li H. Zhuo J. Zhang Y. Wang J. Chen L. (2016). The Human Brainnetome Atlas: A New Brain Atlas Based on Connectional Architecture. *Cereb. Cortex.* 26 3508–3526. 10.1093/cercor/bhw157 27230218 PMC4961028

[B4] Fusconi M. Chistolini A. de Virgilio A. Greco A. Massaro F. Turchetta R. (2012). Sudden sensorineural hearing loss: a vascular cause? Analysis of prothrombotic risk factors in head and neck. *Int. J. Audiol.* 51 800–805. 10.3109/14992027.2012.705904 22928918

[B5] Fuster J. M. (2000). Executive frontal functions. *Exp. Brain Res.* 133, 66–70. 10.1007/s002210000401 10933211

[B6] Haller S. Zaharchuk G. Thomas D. L. Lovblad K. O. Barkhof F. Golay X. (2016). Arterial Spin Labeling Perfusion of the Brain: Emerging Clinical Applications. *Radiology* 281 337–356. 10.1148/radiol.2016150789 27755938

[B7] Hickok G. Poeppel D. (2007). The cortical organization of speech processing. *Nat. Rev. Neurosci.* 8 393–402. 10.1038/nrn2113 17431404

[B8] Hiser J. Koenigs M. (2018). The Multifaceted Role of the Ventromedial Prefrontal Cortex in Emotion. Decision Making, Social Cognition, and Psychopathology. *Biol. Psychiatr.* 83 638–647. 10.1016/j.biopsych.2017.10.030 29275839 PMC5862740

[B9] Husain F. T. Medina R. E. Davis C. W. Szymko-Bennett Y. Simonyan K. Pajor N. M. (2011). Neuroanatomical changes due to hearing loss and chronic tinnitus: a combined VBM and DTI study. *Brain Res.* 1369 74–88. 10.1016/j.brainres.2010.10.095 21047501 PMC3018274

[B10] Job A. Jaroszynski C. Kavounoudias A. Jaillard A. Delon-Martin C. (2020). Functional Connectivity in Chronic Nonbothersome Tinnitus Following Acoustic Trauma: A Seed-Based Resting-State Functional Magnetic Resonance Imaging Study. *Brain Connect* 10 279–291. 32458713 10.1089/brain.2019.0712

[B11] Jones D. T. Graff-Radford J. (2021). Executive Dysfunction and the Prefrontal Cortex. *Continuum* 27 1586–1601. 10.1212/con.0000000000001009 34881727

[B12] Jung da J. Park J. H. Jang J. H. Lee K. Y. (2016). The efficacy of combination therapy for idiopathic sudden sensorineural hearing loss. *Laryngoscope* 126 1871–1876. 10.1002/lary.25751 26972103

[B13] Knight R. T. Hillyard S. A. Woods D. L. Neville H. J. (1981). The effects of frontal cortex lesions on event-related potentials during auditory selective attention. *Electroencephalogr. Clin. Neurophysiol.* 52 571–582. 10.1016/0013-4694(81)91431-0 6172256

[B14] Kuusinen V. Cesnaite E. Peräkylä J. Ogawa K. H. Hartikainen K. M. (2018). Orbitofrontal Lesion Alters Brain Dynamics of Emotion-Attention and Emotion-Cognitive Control Interaction in Humans. *Front. Hum. Neurosci.* 12:437. 10.3389/fnhum.2018.00437 30443211 PMC6221981

[B15] Li X. Zhao P. Qiu X. Ding H. Lv H. Yang Z. (2020). Lateralization Effects on Cerebral Blood Flow in Patients With Unilateral Pulsatile Tinnitus Measured With Arterial Spin Labeling. *Front. Hum. Neurosci.* 14:591260. 10.3389/fnhum.2020.591260 33281587 PMC7705237

[B16] Liu Y. Chen Q. Xu Y. (2020). Research progress in refractory sudden hearing loss: steroid therapy. *J. Int. Med. Res.* 48:300060519889426. 10.1177/0300060519889426 31939327 PMC7254608

[B17] Lomber S. G. Meredith M. A. Kral A. (2010). Cross-modal plasticity in specific auditory cortices underlies visual compensations in the deaf. *Nat. Neurosci.* 13 1421–1427. 10.1038/nn.2653 20935644

[B18] Luan Y. Wang C. Jiao Y. Tang T. Zhang J. Teng G. J. (2019). Prefrontal-Temporal Pathway Mediates the Cross-Modal and Cognitive Reorganization in Sensorineural Hearing Loss With or Without Tinnitus: A Multimodal MRI Study. *Front. Neurosci.* 13:222. 10.3389/fnins.2019.00222 30930739 PMC6423409

[B19] Martín-Fernández J. Burunat I. Modroño C. González-Mora J. L. Plata-Bello J. (2021). Music Style Not Only Modulates the Auditory Cortex, but Also Motor Related Areas. *Neuroscience* 457 88–102. 10.1016/j.neuroscience.2021.01.012 33465413

[B20] Meng Z. Zheng Y. Liu S. Wang K. Kong X. Tao Y. (2012). Reliability and validity of the chinese (mandarin) tinnitus handicap inventory. *Clin. Exp. Otorhinolaryngol.* 5 10–16. 10.3342/ceo.2012.5.1.10 22468196 PMC3314799

[B21] Minosse S. Garaci F. Martino F. Mauro R. D. Melis M. Giuliano F. D. (2020). Global and local reorganization of brain network connectivity in sudden sensorineural hearing loss. *Annu. Int. Conf. IEEE Eng. Med. Biol. Soc.* 2020 1730–1733. 10.1109/embc44109.2020.9175688 33018331

[B22] Morcom A. M. Friston K. J. (2012). Decoding episodic memory in ageing: a Bayesian analysis of activity patterns predicting memory. *Neuroimage* 59 1772–1782. 10.1016/j.neuroimage.2011.08.071 21907810 PMC3236995

[B23] Mores J. T. Bozza A. Magni C. Casali R. L. Amaral M. (2019). Clinical profile and implications of tinnitus in individuals with and without hearing loss. *Codas* 31:e20180029. 10.1590/2317-1782/20192018029 31644709

[B24] Ponticorvo S. Manara R. Pfeuffer J. Cappiello A. Cuoco S. Pellecchia M. T. (2019). Cortical pattern of reduced perfusion in hearing loss revealed by ASL-MRI. *Hum. Brain Mapp.* 40 2475–2487. 10.1002/hbm.24538 30715769 PMC6865742

[B25] Qu H. Tang H. Pan J. Zhao Y. Wang W. (2020). Alteration of Cortical and Subcortical Structures in Children With Profound Sensorineural Hearing Loss. *Front. Hum. Neurosci.* 14:565445. 10.3389/fnhum.2020.565445 33362488 PMC7756106

[B26] Shen K. K. Welton T. Lyon M. McCorkindale A. N. Sutherland G. T. Burnham S. (2020). Structural core of the executive control network: A high angular resolution diffusion MRI study. *Hum. Brain Mapp.* 41 1226–1236. 10.1002/hbm.24870 31765057 PMC7267982

[B27] Shiohama T. McDavid J. Levman J. Takahashi E. (2019). The left lateral occipital cortex exhibits decreased thickness in children with sensorineural hearing loss. *Int. J. Dev. Neurosci.* 76 34–40. 10.1016/j.ijdevneu.2019.05.009 31173823 PMC6698225

[B28] St Lawrence K. S. Wang J. (2005). Effects of the apparent transverse relaxation time on cerebral blood flow measurements obtained by arterial spin labeling. *Magn. Reson. Med.* 53 425–433. 10.1002/mrm.20364 15678532

[B29] Stachler R. J. Chandrasekhar S. S. Archer S. M. Rosenfeld R. M. Schwartz S. R. Barrs D. M. (2012). Clinical practice guideline: sudden hearing loss. *Otolaryngol. Head Neck Surg.* 146 S1–S35. 10.1177/0194599812436449 22383545

[B30] Tervaniemi M. Hugdahl K. (2003). Lateralization of auditory-cortex functions. *Brain Res. Brain Res. Rev.* 43 231–246. 10.1016/j.brainresrev.2003.08.004 14629926

[B31] Xia S. Song T. Che J. Li Q. Chai C. Zheng M. (2017). Altered Brain Functional Activity in Infants with Congenital Bilateral Severe Sensorineural Hearing Loss: A Resting-State Functional MRI Study under Sedation. *Neural Plast.* 2017:8986362. 10.1155/2017/8986362 28255465 PMC5309418

[B32] Xu H. Fan W. Zhao X. Li J. Zhang W. Lei P. (2016). Disrupted functional brain connectome in unilateral sudden sensorineural hearing loss. *Hear Res.* 335 138–148. 10.1016/j.heares.2016.02.016 26969260

[B33] Yang M. Chen H. J. Liu B. Huang Z. C. Feng Y. Li J. (2014). Brain structural and functional alterations in patients with unilateral hearing loss. *Hear Res.* 316 37–43. 10.1016/j.heares.2014.07.006 25093284

[B34] Zhang G. Y. Yang M. Liu B. Huang Z. C. Chen H. Zhang P. P. (2015). Changes in the default mode networks of individuals with long-term unilateral sensorineural hearing loss. *Neuroscience* 285 333–342. 10.1016/j.neuroscience.2014.11.034 25463518

[B35] Zou Y. Ma H. Liu B. Li D. Liu D. Wang X. (2021). Disrupted Topological Organization in White Matter Networks in Unilateral Sudden Sensorineural Hearing Loss. *Front. Neurosci.* 15:666651. 34321993 10.3389/fnins.2021.666651 PMC8312563

[B36] Zung W. W. (1971). A rating instrument for anxiety disorders. *Psychosomatics* 12 371–379. 10.1016/s0033-3182(71)71479-0 5172928

[B37] Zung W. W. (1972). The Depression Status Inventory: an adjunct to the Self-Rating Depression Scale. *J. Clin. Psychol.* 28 539–543. 10.1002/1097-4679(197210)28:4<539::aid-jclp2270280427<3.0.co;2-s 5080837

